# Photon-in photon-out hard X-ray spectroscopy at the Linac Coherent Light Source

**DOI:** 10.1107/S1600577515004488

**Published:** 2015-04-15

**Authors:** Roberto Alonso-Mori, Dimosthenis Sokaras, Diling Zhu, Thomas Kroll, Mathieu Chollet, Yiping Feng, James M. Glownia, Jan Kern, Henrik T. Lemke, Dennis Nordlund, Aymeric Robert, Marcin Sikorski, Sanghoon Song, Tsu-Chien Weng, Uwe Bergmann

**Affiliations:** a Linac Coherent Light Source, SLAC National Accelerator Laboratory, 2575 Sand Hill Road, Menlo Park, CA 94025, USA; b Stanford Synchrotron Radiation Lightsource, SLAC National Accelerator Laboratory, 2575 Sand Hill Road, Menlo Park, CA 94025, USA; c Physical Biosciences Division, Lawrence Berkeley National Laboratory, Berkeley, CA 94720, USA

**Keywords:** FEL, hard X-ray emission spectroscopy, XES, XRS

## Abstract

X-ray free-electron lasers (FELs) have opened unprecedented possibilities to study the structure and dynamics of matter at an atomic level and ultra-fast timescale. Many of the techniques routinely used at storage ring facilities are being adapted for experiments conducted at FELs. In order to take full advantage of these new sources several challenges have to be overcome. They are related to the very different source characteristics and its resulting impact on sample delivery, X-ray optics, X-ray detection and data acquisition. Here it is described how photon-in photon-out hard X-ray spectroscopy techniques can be applied to study the electronic structure and its dynamics of transition metal systems with ultra-bright and ultra-short FEL X-ray pulses. In particular, some of the experimental details that are different compared with synchrotron-based setups are discussed and illustrated by recent measurements performed at the Linac Coherent Light Source.

## Abstract 2

A description of hard X-ray photon-in photon-out spectroscopy techniques for X-ray free electron laser applications is given. A discussion of the instrumentation suitable for taking full advantage of these new sources and a description of recent measurements performed and related examples are also presented.

## 1. Introduction   

X-ray free-electron lasers (FELs), such as the Linac Coherent Light Source (LCLS) (Emma *et al.*, 2010 ▶) at SLAC National Accelerator Laboratory, are fourth-generation X-ray sources operating on the principle of self-amplified spontaneous emission (SASE), providing extremely bright ultra-short (fs) coherent X-ray pulses. In order to take full advantage of some of the X-ray techniques that have become routine in the last decades from synchrotron radiation (SR) storage ring based research, these techniques have to be adapted and modified to the unique characteristics of these new FEL sources. In particular one needs to consider X-ray beam characteristics related to the stochastic nature of the pulses from the SASE process as well as other properties that are different from those of SR sources. These include pulse energy, bandwidth, profile (spatial, temporal and spectral), repetition rate and shot-to-shot spectral and intensity fluctuations. For example, the number of photons in a single FEL pulse is more than six orders of magnitude higher than in a typical SR pulse. This requires very different methods of sample delivery, as well as data collection and detection, where each shot is treated as its own separate experiment (as compared to integrating many pulses, the common practice at SR sources). These ultra-bright pulses can also induce non-linear effects, including multiple electron ionizations (Young *et al.*, 2010 ▶) and stimulated emission processes (Rohringer *et al.*, 2012 ▶). They can also outrun radiation damage on sensitive samples caused by radical diffusion (since the time scale for radical diffusion is in the picosecond range), a common problem in SR sources, particularly with biological samples (Yano *et al.*, 2005 ▶). Hard X-ray spectroscopy offers several practical advantages over its soft X-ray counterpart, in particular related to the sample environment and the use of Bragg optics for detection, which has broadened the variety and conditions of samples that can be probed. In this paper we will discuss how hard X-ray photon-in photon-out spectroscopy is used with FEL sources to study the electronic structure and its dynamics in various systems.

Photon-in photon-out X-ray spectroscopy typically refers to techniques where the photon-out channel is measured with high spectral and/or momentum resolution. Non-resonant X-ray emission spectroscopy (XES), where the photo-excitation is well above the absorption edge of the element under investigation, can probe the effective spin of 3*d* transition metal ions (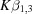 and 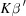) as well as the metal bonding orbitals (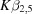 and 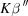) and has been used to investigate a variety of systems including metalloproteins (Bergmann *et al.*, 1998 ▶; Messinger *et al.*, 2001 ▶; Pushkar *et al.*, 2010 ▶; Lancaster *et al.*, 2011 ▶), coordination complexes (Visser *et al.*, 2001 ▶; Smolentsev *et al.*, 2009 ▶; Delgado-Jaime *et al.*, 2011 ▶) and geochemical systems (Badro *et al.*, 2004 ▶; Bargar *et al.*, 2005 ▶). In resonant inelastic X-ray scattering (RIXS), also known as resonant X-ray emission spectroscopy (RXES) (Kotani & Shin, 2001 ▶), the incident energy is tuned to resonantly excite an element at its absorption edge and the emitted/scattered X-ray spectrum is recorded at each excitation energy. This method has seen a large growth in the last few years and proved to be useful for studying different systems (see, for example, Glatzel *et al.*, 2002*a* ▶; de Groot *et al.*, 2007 ▶; Ament *et al.*, 2011 ▶; Booth *et al.*, 2012 ▶; Lundberg *et al.*, 2013 ▶; Kroll *et al.*, 2014 ▶). In high-energy-resolution fluorescence detected (HERFD) XANES (Hämäläinen *et al.*, 1991 ▶; Friebel *et al.*, 2011 ▶) the emission energy is fixed while the incident energy is scanning across an absorption edge. This method is experimentally less elaborate than RIXS and has been used to study unoccupied electronic states with a higher energy resolution as compared with conventional XANES due to the suppression of the core-hole lifetime broadening (Heijboer *et al.*, 2004 ▶; Safonova *et al.*, 2006 ▶; Glatzel *et al.*, 2009 ▶). In the case of extended X-ray absorption fine structure (EXAFS), high-resolution detection has been used to enhance the spatial resolution by either discriminating against the fluorescence signal from unwanted elements (Pushkar *et al.*, 2007 ▶; Glatzel *et al.*, 2005 ▶) or by selecting a specific chemical site in mixed-valent systems (Glatzel *et al.*, 2002*b* ▶). Using the inelastic scattering of hard X-rays by core electrons, or X-ray Raman scattering (XRS), energy-loss information equivalent to direct X-ray absorption spectroscopy (XAS) can be obtained when measured in the low momentum transfer regime (Bergmann *et al.*, 2000 ▶). Furthermore, electric non-dipole contribution, not obtainable with conventional XAS techniques, can be studied with XRS at high momentum transfer (Krisch *et al.*, 1997 ▶; Sternemann *et al.*, 2003 ▶; Soininen *et al.*, 2005 ▶). Moreover, what makes XRS a powerful technique is the fact that low-*Z* atomic species can be probed with hard X-rays. This has been successfully used to study the bulk properties of low-*Z* systems under ambient and extreme conditions (Wernet *et al.*, 2004 ▶; Mao *et al.*, 2006 ▶; Bergmann *et al.*, 2007 ▶), including oxygen *K*-edge EXAFS on water using the XRS technique (Bergmann *et al.*, 2007 ▶). For a more detailed review of all the hard X-ray techniques described above, see Bergmann *et al.* (2002 ▶) and Glatzel & Bergmann (2005 ▶).

The element-specific structural and chemical sensitivity of these techniques, as well as their short core-hole lifetimes (∼fs), makes them very powerful tools for the study of ultra-fast dynamics and other processes that rely on ultra-short X-ray pulses. In order to take full advantage of sources that provide such pulses, in particular the ultra-bright FEL sources, technical adaptations and in some cases new experimental methods of implementing these techniques are required. SR-based photon-in photon-out hard X-ray spectroscopy experiments are typically performed by scanning either the incident X-ray energy (XRS, HERFD, selective XAS) or the emitted X-ray energy (XES) or both (RIXS). Any spectroscopy based on scanning methods requires the exact knowledge of the incident photon flux in order to properly normalize the signal and avoid spectral distortions. SASE FEL sources are stochastic by nature, resulting in large pulse-to-pulse intensity fluctuations up to 100%. Furthermore, their spectral width is on the order of 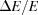 ≃ 10^−2^ and pulse-to-pulse spectral fluctuations makes the use of FELs challenging for spectroscopic techniques that require a highly monochromatic incident beam (typically 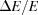 ≃ 10^−4^ or better). For non-resonant XES, the incident X-ray energy and bandwidth is not critical as long as it is above the absorption edge of the element under study and the photon energy of the incident X-rays is not varied. In the case of HERFD, RIXS and XRS (when using a scanning analyzer) a monochromatic incident beam that is scanned in energy is required. In typical SASE FEL operations this requires an incident beam monochromator.

The challenges of using a monochromator at a FEL are twofold: (i) the loss in photon flux, since only a few percent of the SASE pulse is transmitted through the monochromator, and (ii) the very large intensity fluctuations of up to 100% due to the stochastic nature of the SASE pulses (Lee *et al.*, 2012 ▶). Recently, it has been shown that hard X-ray FELs can also operate in a more monochromatic self-seeding mode (Amann *et al.*, 2012 ▶). This improves the bandwidth up to 2 × 10^−4^ 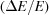; however, a strong SASE background is present in this mode and a monochromator to filter out this unwanted background is used. An increase in the stability of the klystron electrical power supply units is expected to improve the performance of self-seeding mode in the future. Even with such improvements the existence of an accurate normalization protocol is critical to obtain a reliable spectrum. Moreover, the thermal load on the monochromator crystals can result in spatial and spectral drifts, potentially inducing other experimental challenges such as pump/probe and target/X-ray overlap, particularly when using a focused beam. Although the FEL facilities are continuously improving the high-heat-load X-ray optics, beam diagnostics and normalization tools, it is clear from this discussion that X-ray spectroscopy instrumentation that does not depend on scanning and/or the exact shot-by-shot pulse characterization has the advantage of reducing systematic errors. As will be discussed in this paper, wavelength-dispersive photon-out spectrometers and monochromatic analyzers used at fixed energies fall into this class of instrumentation. We will discuss recent examples of photon-in photon-out measurements performed at the LCLS. These include the demonstration that XES can be used in combination with the LCLS X-ray beam and provide information on the intact electronic structure of redox active compounds (Alonso-Mori *et al.*, 2012*a* ▶), studies of the charge transfer and spin dynamics in coordination complexes (Zhang *et al.*, 2014 ▶) and of the redox state of photoactive proteins (Kern *et al.*, 2013 ▶, 2014 ▶). While we will focus on work performed or underway at LCLS, the insights we discuss are applicable more broadly to other ultra-fast X-ray sources and could also stimulate some future work at SR sources.

## 2. Instrumentation   

### 2.1. Spectrometers   

**Figure 1:**
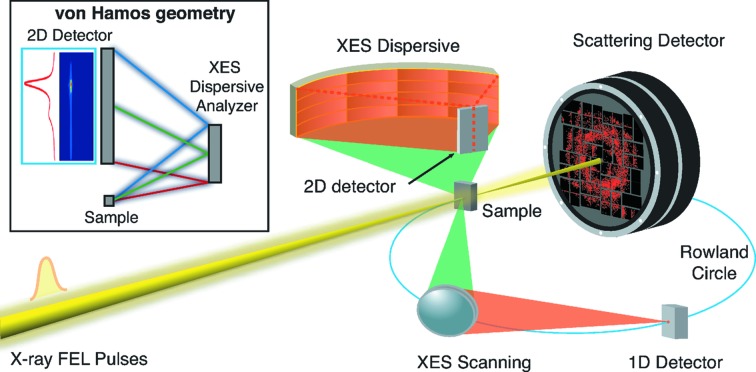
Schematic for simultaneous collection of photon-in photon-out and forward-scattering signals. The dispersive von Hamos XES spectrometer focuses the spectrum on a two-dimensional detector placed above the intersection point and the Rowland XES spectrometer can be tuned to collect different emission lines concurrently while obtaining data from a complementary scattering technique. Inset: vertical cut of the dispersive von Hamos geometry with a crystal analyzer and a two-dimensional detector.

Analysis of scattered or emitted hard X-rays is typically performed by Bragg diffraction from single or multiple perfect-crystal analyzers capable of collecting a large solid angle (up to few % of 4π) with high energy resolution. At SR sources, Rowland-circle Johann-type spectrometers, which use monochromatic analyzers, are most commonly used (Stojanoff *et al.*, 1992 ▶; Sokaras *et al.*, 2013 ▶). These spectrometers are operated in point-to-point focusing geometry, offering the best signal-to-background ratio. Another approach is to use polychromatic analysers based on dispersive geometries, where the X-rays are spatially discriminated onto a position-sensitive two-dimensional detector. Such spectrometers allow the collection of an extended spectral range in a single shot, or after integrating multiple shots when the signal intensity is low, with no need of scanning optics or saving one scanning dimension when the incident energy has to be varied (as for collecting a RIXS plane or an XRS spectrum). This results in the elimination of the scanning dead-time as well as of any possible artifact introduced by scanning synchronization issues. Dispersive spectrometers have simpler and more robust mechanics, and therefore enhanced mechanical stability. Additionally, the crystal alignment protocol is relatively simple as compared with a Johann spectrometer, since the signal is monitored by a two-dimensional detector. Most importantly, the XES spectrum does not necessarily need to be normalized to the incoming flux since the intensities for all energies are recorded simultaneously. This is particularly suited for time-resolved measurements such as pump–probe experiments and for overcoming the problem of the pulse fluctuations when used with a FEL beam. Similarly, the use of a dispersive geometry can also help to avoid the inherent problems caused by inhomogeneities of the sample, which is a common issue at FELs where the sample usually has to be replenished after each shot, either by using a liquid jet or by rastering an extended solid sample. It is also specially well suited to be combined with other techniques, such as XRD or other scattering methods, to simultaneously obtain both electronic and atomic structural information for each pulse (see Fig. 1 ▶).

There are several approaches to dispersive analyzer geometries including flat crystals or various forms of curved crystals. Arguably the most straightforward approach is the von Hamos geometry (von Hamos, 1932 ▶). Here, the analyzer crystals are cylindrically bent along the non-dispersive direction and diffract and focus the emitted radiation from the sample to a two-dimensional detector following Bragg’s law, 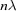 = 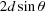. The sample and detector are positioned on the axis of curvature of the crystal analyzers. The height of the crystals and their position relative to the interaction point of beam and sample defines the range of Bragg angles and corresponding energies (see Fig. 1, left inset ▶). The curvature direction of the analyzers provides focusing, whereas the perpendicular direction gives the energy dispersion. For each crystal analyzer, integration along the focusing direction of the signal on the two-dimensional detector results in a spectrum. In order to cover the whole spectral range, the detector active area in the dispersive direction is required to be twice the height of the crystal analyzers, which is chosen to match the energy range of the emission lines of interest. Small detector pixel size is also convenient for achieving better spatial and therefore energy resolution. For example, the multi-crystal von Hamos spectrometer currently available at LCLS (Alonso-Mori *et al.*, 2012*b* ▶), composed of 16 crystal analyzers bent to 500 mm, is operated with a 110 µm × 110 µm pixel size detector corresponding to 0.14 eV resolution per pixel for Fe *K*β at 7057 eV (or 0.06 eV for Mn *K*β at 6490 eV). The spectrometer is used at Bragg angles close to backscattering in order to optimize the solid angle per energy unit and the energy resolution. An additional advantage derived from the use of cylindrically bent crystals is the smaller strain introduced in the lattice plains as compared with spherically bent crystals, resulting in better intrinsic energy resolution and reflectivity.

Various factors have to be taken into account when choosing the geometry of the spectrometer for a particular application. The most critical are energy resolution, solid angle and energy range. In order to be sensitive to chemical changes, the energy resolution should be better than the core level lifetime broadening of the emission line under study, typically tenths of to a few eV. This requirement drives the design of the spectrometer in terms of Bragg angle, radius of curvature and detector pixel size for a given beam size. In a dispersive spectrometer the solid angle per energy unit can be approximated by the ratio of the total solid angle and the captured energy range where both have a strong dependence on the Bragg angle (θ) with the back scattering (θ ≃ 90°) resulting in the largest solid angle per eV. Therefore the most efficient von Hamos spectrometers are operated above 75° Bragg angles. Such large Bragg angles are also beneficial considering the energy range of a few tens of eV typically required to collect a spectrum. The instrument at LCLS, for example, uses crystals with 500 mm bending radius and 25 mm height capturing an energy range of 30 eV at 6.5 keV when used at 84°. There are cases where a dispersive setup is not practical because either the Bragg angle is too far from back-scattering or the solid angle corresponding to the required energy range is too small. In such cases it is advantageous to use monochromatic spectrometers based on the Rowland geometry. Examples are studies of transient states at a specific spectral feature, or HERFD and site-selective XAS studies, where the incident energy is scanned at a fixed analyzer energy. Moreover, the use of a Rowland geometry is advantageous for some cases where the signal is very weak compared to the background, as the dispersive setups collect the solid angle into an extended detector area in contrast to the point-to-point focusing Rowland approach. Finally, in some time-resolved applications, it is beneficial to use both instrumentation types simultaneously. For example, the dispersive setup is used to collect a full *K*β_1,3_ spectrum to study the time dependence of a chemical change, while the Rowland-based spectrometer tracks the stronger *K*α_1_ peak as a time-zero reference. Moreover, photon-in photon-out spectroscopy measurements can be combined with other techniques such as X-ray scattering or diffraction to simultaneously gain information on the geometric structure of the system under study (see Fig. 1 ▶).

In summary, the appropriate instrumentation has to be evaluated for each photon-in photon-out spectroscopic technique, based on the arguments described above and the FEL beam characteristics:

(i) Non-resonant XES has the least stringent requirements for FEL-based studies, as the energy fluctuations and spectral width of the incident FEL pulse are not critical. Collecting an XES spectrum with a wavelength-dispersive instrument in a shot-by-shot manner further circumvents normalization issues associated with the intensity fluctuations.

(ii) For RIXS applications where typically a monochromatic and tunable incident beam is required, good shot-by-shot normalization is critical. However, a dispersive analyzer can simplify the data collection and remove the photon-out scanning dimension.

(iii) For HERFD and other selective XAS, only a narrow bandwidth of the emitted photons is of interest. Here the best choice are spherically bent crystal analyzers in the Rowland geometry operated at a fixed energy. They have the best signal to noise and a large solid angle. Note that, as in RIXS, the challenges associated with scanning the incident beam persist.

(iv) XRS is a very weak non-resonant scattering process (∼ few Barns) where the X-ray energy loss is measured. Conceptually two methods can be used: (i) varying the incident photon energy while measuring the intensity of the scattered photons at a fixed energy, (ii) measuring the energy-loss spectrum at a fixed incident energy. In either case a monochromatic X-ray beam is required (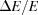 ≃ 10^−4^). Which setup is preferred depends on the experimental specifics such as the spectral range of interest, sample concentration and the expected spectral changes. We note that, when using a dispersive analyser, the XRS method can avoid scanning of the incident beam and its related difficulties.

### 2.2. Detectors   

The selected spectrometer design drives the detector requirements for a particular experiment. In general, as for any spectroscopic application, the readout noise is critical, especially for collecting weak signals, and defines the lower threshold of the detectable signal. In particular, for most FEL-based spectroscopy experiments, the readout speed of the detector has to match the repetition rate of the machine [*e.g.* tenths to hundreds of Hz for machines based on klystron technology like LCLS or SACLA (Ishikawa *et al.*, 2012 ▶) and >kHz for future machines based on superconducting technology like LCLS II or European XFEL (XFEL, 2006 ▶)]. Moreover, the detector needs to be able to handle the large number of photons arriving in very short pulses (fs range). The commonly used detectors that fulfil the requirements can be divided in two main classes, point detectors for Rowland instruments using monochromatic crystals and two-dimensional detectors for use in conjunction with dispersive setups.

Non-position-resolving detectors for Rowland instruments are usually based on solid-state photon-counting technology. In detectors, the electron/hole pair charge, created by the absorption of the incident X-ray and accumulated through an applied potential difference, is carried to the electronics for readout and correlated to the energy and intensity of the incident X-ray photon. Such detectors provide simultaneous detection for a wide energy range of X-rays with a great detection efficiency (up to 100% for 4–10 keV) and energy resolution (∼135 eV at 5900 eV). During the last decade, solid-state-based detectors have been upgraded with the superior silicon drift technology, whose main advantage is their short shaping time leading to very high count rates (several millions of events per second). However, at a FEL, where a large number of photons arrive at the sample in very short pulses, the emitted signal can only be energy discriminated by the detector if it is of the order of one to a few photons per pulse. For a large fraction of the FEL spectroscopic applications where the detector is only used to count the emitted X-ray signal, simpler detectors such as photodiodes and avalanche photodiodes would fulfill the requirements.

To spatially discriminate the range of energies simultaneously diffracted by a dispersive instrument requires a position-resolving, or two-dimensional, detector. Currently, there are limited options available that fulfil the requirements for FEL applications, especially in terms of fast readout speed. This is mainly related to the difficulty of reading out the charge of the relatively large area necessary for most of the applications (the energy range to be collected by the detector is usually tens of eV which spatially translates to several centimetres along the dispersive direction). An interesting option, based on the development of direct conversion fully depleted CCDs, is the pnCCD sensor (Strüder *et al.*, 2010 ▶), primarily targeting astronomy and time-resolved X-ray applications. The pnCCD readout is fully column parallel and uses dedicated multichannel microelectronics for fast readout speeds up to multiple hundred frames per second (depending on the CCD size). Similar solutions are implemented by the recent LBNL fCCD (Doering *et al.*, 2011 ▶) and SACLA’s MPCCD (Hatsui, 2014 ▶) detector designs. Hybrid detectors are also a suitable two-dimensional technology for FEL spectroscopic applications. Among these, the photon-counting approach (*e.g.* Pilatus and Medipix) is incompatible with the FEL X-ray beam, since the intensity yielded by all photons from the very short pulse are treated as a single signal unit by these type of detectors. Another category of hybrid detectors, specifically developed for FEL applications, uses the charge integrating approach, where the photon-induced charge is continuously integrated. This technology is used in the CSPAD (Herrmann *et al.*, 2013 ▶) family of detectors developed for the LCLS. In these, the preamplifier and amplifier are condensed within the pixel size and the readout speed is designed to match that of the LCLS machine (usually hundreds of Hz). This results in an increased readout noise limiting the energy resolution to ∼3000 eV. Near future designs, like the ePix family (Dragone *et al.*, 2014 ▶), are being developed and planned to achieve <1000 eV resolution using similar technology as in the current CSPAD detectors. A review of LCLS detectors is given by Blaj *et al.* (2015) ▶. A similar approach was used or is being used for the development of several other fast readout detectors for XFEL applications, AGIPD for the European XFEL (Henrich *et al.*, 2000 ▶) and Jungfrau for Swissfel (Mozzanica *et al.*, 2014 ▶).

The development of detectors with very high energy resolution (*e.g.* superconducting detectors based on the calorimetric detection of the quantum energy of the incident photon), in the sub-eV to few eV range, could render the use of spectrometers based on optics unnecessary for some spectroscopy applications in the future. This would greatly facilitate measurements based on photon-in photon-out spectroscopy techniques providing exceptional energy resolution, and still attaining large solid angles by placing the detector close to the source. For this to become a reality reduction of the costs and improvements in the technology leading to better performance, in particular better efficiency for hard X-rays (above 1 keV), larger active area and higher dynamic range and readout speed for FEL applications, is necessary (Ullom *et al.*, 2014 ▶).

## 3. Applications   

**Figure 2:**
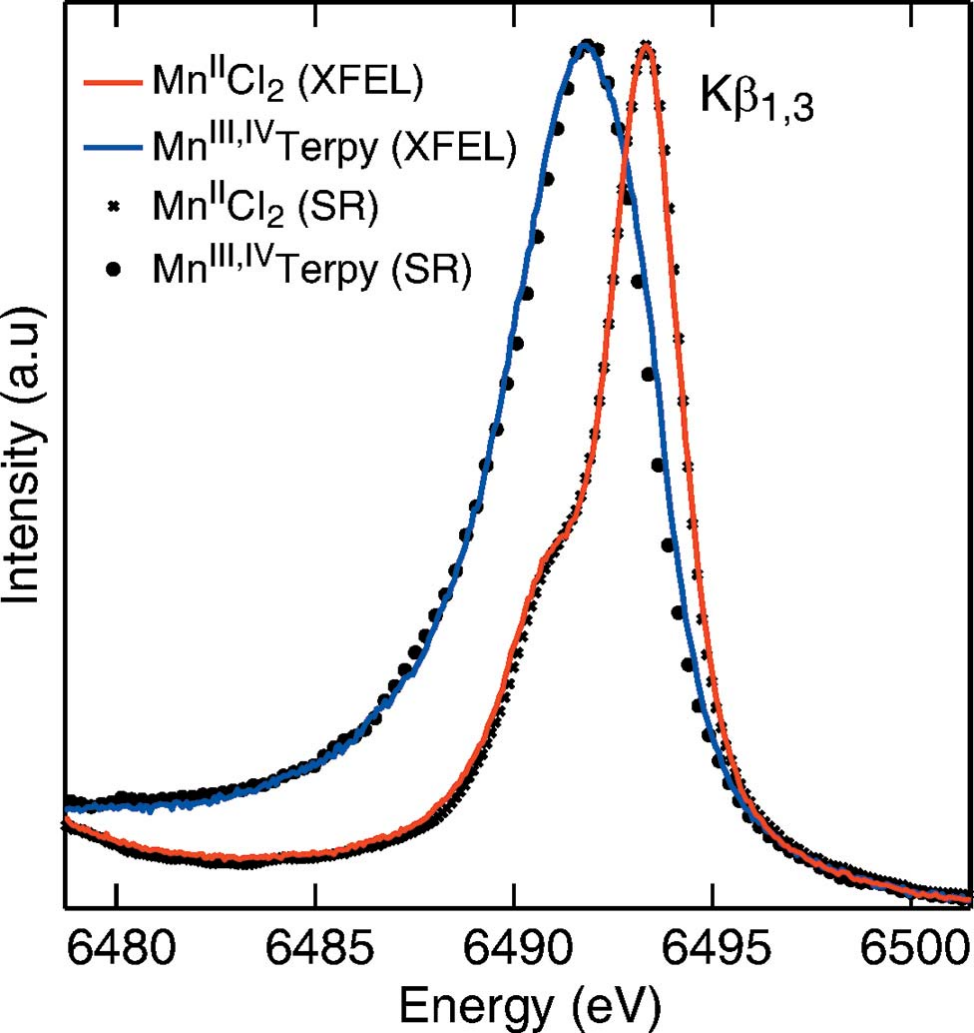
Mn 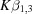 spectra from Mn^II^Cl_2_ (red) and redox-sensitive 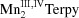 (blue) collected at the CXI instrument. The symbols show the spectra from the Mn^II^ and 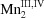 complexes collected with a similar setup at SSRL beamline 6-2 for comparison purposes. Figure reprinted with permission from Alonso-Mori *et al.* (2012*a* ▶).

With the advent of FELs, there was the question of whether the electronic structure could be probed in X-ray spectroscopy experiments before becoming affected by the intense X-ray pulses. X-ray-induced changes in the electronic structure would translate into altered intensities and/or spectral shapes. To evaluate this question, Alonso-Mori *et al.* (2012*a* ▶) measured the Mn 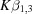 X-ray emission spectra of two redox-sensitive Mn complexes in aqueous solutions (Mn^II^Cl_2_, 500 m*M* and 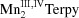, 180 m*M*), using the 1.5 µm focus at the CXI instrument (Liang *et al.*, 2015 ▶). The incident X-rays were tuned to 50 and 100 fs pulse length at 7 and 9.5 keV. A dispersive spectrometer based on the von Hamos geometry, populated with 16 Si(440) crystal analyzers, was used to record the emission spectra (Alonso-Mori *et al.*, 2012*b* ▶). The spectra were found to be in agreement with data from undamaged samples collected at low dose using SR, as dispalyed in Fig. 2 ▶. This study demonstrates that, under given experimental conditions, information from the electronic structure of dilute redox-active transition metal compounds can be obtained at a FEL using XES.

**Figure 3:**
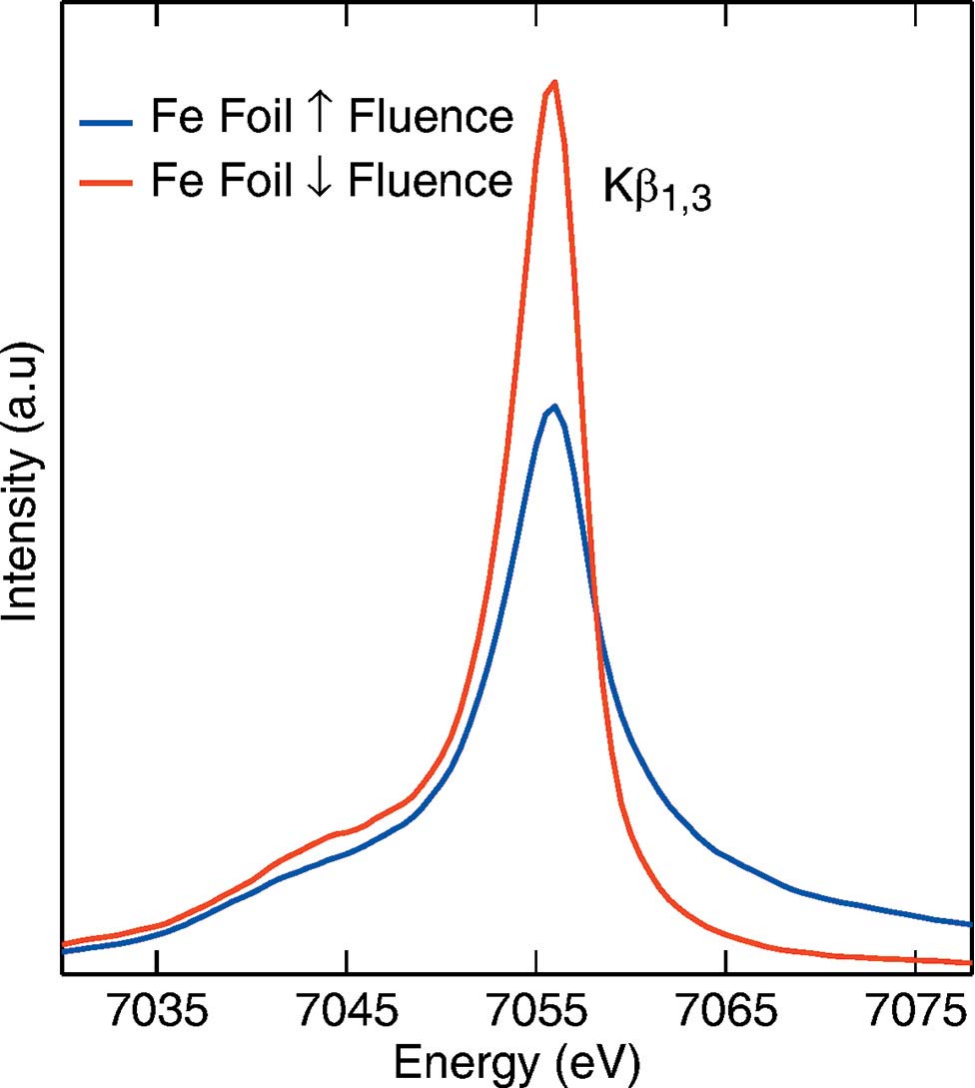
Fe metal 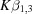 spectra collected at the XPP instrument. The blue curve was recorded with unfocused beam. The red curve was recorded with the beam being tightly focused.

However, this is not necessarily the case for every sample and for the whole range of X-ray properties available at a FEL in terms of fluence, energy or pulse length. Fig. 3 ▶ shows two Fe 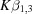 spectra from an Fe metal foil collected by the dispersive von Hamos spectrometer populated by four Ge(620) crystal analyzers. The measurement performed under low-fluence conditions, using an unfocused FEL beam, yielded a spectrum identical to what one would measure at SR sources. However, the spectrum collected under high-fluence conditions with the beam focused down to ∼1.5 µm × 1.5 µm shows clear broadening and a shift of the spectral weight. Both spectra were normalized to the incident flux and were collected with 50 fs pulse length at the XPP instrument (Chollet *et al.*, 2015 ▶). This is a direct indication that, at large fluences easily achievable at FELs, care has to be taken in this type of experiment, especially when measuring concentrated samples.

**Figure 4:**
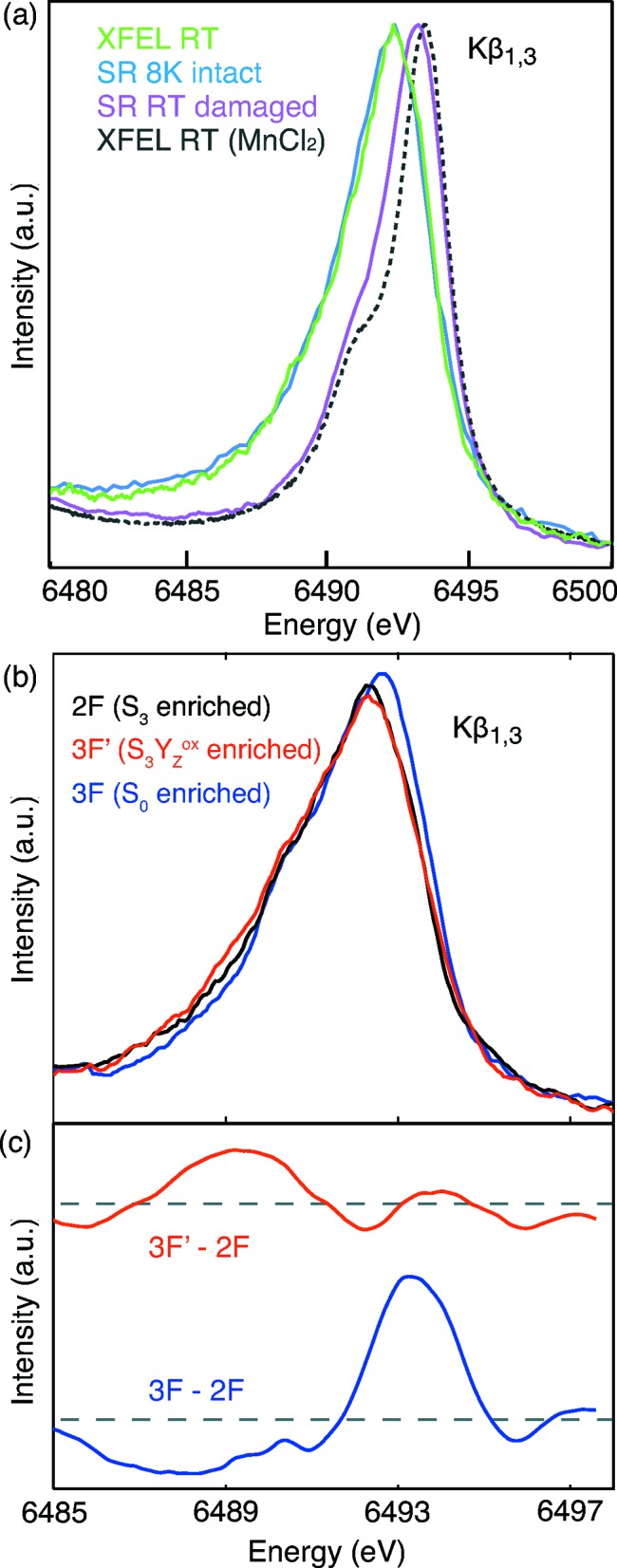
(*a*) Mn 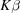 XES spectra of PSII measured in the dark stable S_1_ state at the CXI instrument at RT (green), at a synchrotron at 8 K (blue) and at a synchrotron at RT (pink) in comparison with the spectrum of MnCl_2_ (grey). (*b*) RT XES of PSII in three different states of the photosynthetic cycle measured at CXI. (*c*) Differences between the LCLS Mn 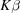 XES of PSII in the 2F and the transient 3F′ state (red) and in the 2F and 3F state (blue). Before calculating the difference curves, spectra were smoothed by cubic polynomial fitting. Figure reprinted with permission from Kern *et al.* (2013 ▶, 2014 ▶).

An example of the advantages of using an FEL to measure low-concentrated highly sensitive samples is the catalytic cluster in the Photosystem II (PSII) complex, which plays a central role in the O_2_ formation in water splitting. The catalytic site in PSII is comprised of a Mn_4_CaO_5_ cluster with the Mn in its dark stable resting state being in oxidation state III and IV (Yano & Yachandra, 2014 ▶). Due to its high susceptibility to X-ray induced damage (Yano *et al.*, 2005 ▶), determining its undamaged electronic and geometric structure at room temperature (RT) using SR sources is very challenging. Instead, the use of the fs pulses of a FEL offers a different route to probe its structure before it is altered by the X-ray photons. As 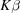 XES is a direct probe of the oxidation state of the metal ion and the excitation energy can be chosen to be compatible with simultaneous X-ray diffraction, a combined setup was developed allowing Mn 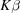 XES on solutions and crystals of PSII to be collected at the CXI instrument. The first XES spectrum of a metalloenzyme obtained at LCLS [*cf*. Fig. 4(*a*) ▶] clearly showed that the electronic structure of the Mn_4_CaO_5_ cluster was not altered within the <50 fs X-ray pulses (and a fluence of ∼2 × 10^−3^ photons Å^−2^ pulse^−1^, corresponding to 500 MGy) used in the measurement (Kern *et al.*, 2013 ▶). The spectra were obtained in about 1 h of measurement time using the dispersive von Hamos spectrometer populated by 16 Si(440) crystal analyzers, a CSPAD-140k detector and an electro-spinning jet (Sierra *et al.*, 2012 ▶) for replenishing the sample for each shot.

After demonstrating the feasibility of this approach it was also possible to monitor the light-induced change in the oxidation state of the catalytic site of PSII at RT using the same setup (Kern *et al.*, 2014 ▶). In the transition from the highest stable oxidized state obtained after illuminating the sample with two light flashes spaced by about 0.5 s (2F,S_3_ state) to the most reduced state (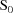), obtained by three consecutive light flashes (3F), a clear reduction of Mn was observed [*cf*. Figs. 4(*b*) and 4(*c*) ▶]. This transition is thought to be from a 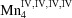 to a 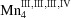 state and the observed shift in the first moment of the Mn 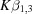 line is in accordance with this oxidation state change. Furthermore, a transient state 250 µs after the third light flash was probed. At this time the transition from the 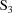 to the 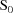 state only started (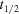 ≃ 1.2–1.5 ms). From the position of the 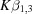 line it was possible to deduce that no Mn reduction has occurred yet, in agreement with other previous spectroscopic studies, indicating a lag phase of about 200 µs before the onset of Mn redox chemistry in this transition (Haumann *et al.*, 2005 ▶; Noguchi *et al.*, 2012 ▶). These studies paved the ground for successful application of 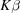 XES of transition metal ions from low-concentrated/biological samples at FELs.

**Figure 5:**
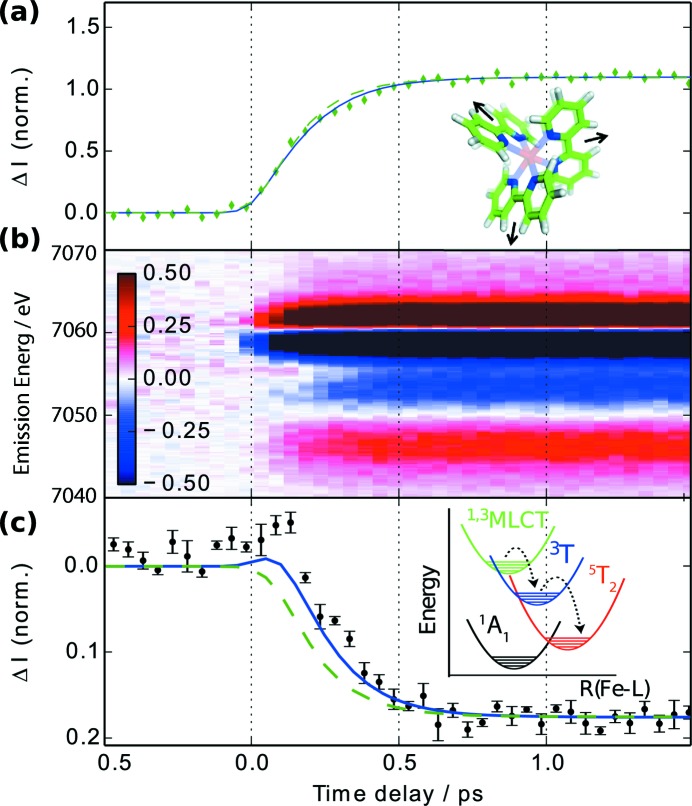
Time-dependent photo-induced Fe 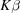 difference spectra (*b*) and kinetic modelling of spin crossover dynamics [(*a*) and (*c*)] for Fe(bpy)_3_. After excitation into the metal–ligand charge transfer state, transition into the HS ^5^T_2_ state [inset in (*c*)] causes an increase of the Fe-to-ligand distance [inset in (*a*)]. The difference signal measured at a 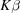 fluorescence energy of 7061 eV [(*a*) green diamonds] and 7054 eV [(*c*) black symbols]. The data have been overlaid with results from global fits including (blue lines) and excluding (green dashed lines) population of and intermediate ^3^T_2_ triplet state. Courtesy of Zhang *et al.* (2014) ▶.

Light-induced charge transfer processes in molecules form the precursor states for real charge separation, which can technically be exploited for energy conversion from optical light in dye-sensitized solar cells. The very limited abundance of the central elements of the most effective molecular systems for this applications (ruthenium or rhenium) has led to extensive research on other transition metal molecular complexes. A deeper understanding of ultra-fast electronic and nuclear transitions of other elements can help here to design cheaply available molecules with efficient charge transfer properties. The Fe complex iron-trisbipyridine [Fe(bpy)_3_] has previously served as such a model system, where an optically excited metal-to-ligand charge transfer state is depleted within less than 200 fs to a high-spin (HS) state. During the process the total spin moment of the Fe atom undergoes a transition from singlet (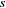 = 0) to quintet state (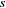 = 2), and a population of an intermediate triplet state is highly debated. The Fe 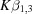 emission line was used at the XPP instrument (Chollet *et al.*, 2015 ▶) to investigate the spin state of Fe(bpy)_3_ (Zhang *et al.*, 2014 ▶). The changes in the emission lines were recorded by means of the dispersive von Hamos spectrometer populated with four Ge(620) crystal analyzers as a function of the pump/probe time delay. The transient spectra from the photoexcited Fe(bpy)_3_ complex in solution are shown in Fig. 5(*b*) ▶. The overall shape of the 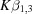 emission is independent of the chemical environment given by the ligand. Static state reference spectra of Fe^II^ in the different spin configurations were used to deconvolute the signal [*cf*. Figs. 5(*a*) and 5(*b*) ▶]. This indicates the existence of an intermediate triplet state, and demonstrates that femtosecond-resolution XES can track the spin crossover dynamics on photoinduced metal-to-ligand charge transfer excitation processes.

**Figure 6:**
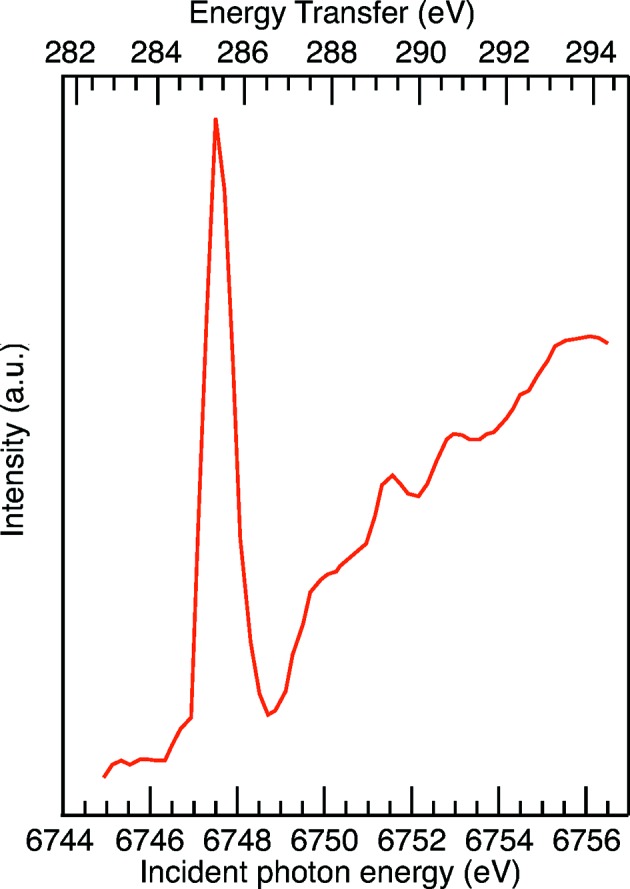
Carbon *K*-edge X-ray Raman spectrum from a polycarbonate film collected at the XPP instrument of the LCLS using SSRL’s 40-crystal X-ray Raman spectrometer, with an integration time of 3 s (360 pulses) per energy point. Courtesy of Sokaras *et al.* (2015) ▶.

The first X-ray Raman experiment was recently performed at the XPP instrument (Sokaras *et al.*, 2015 ▶). Fig. 6 ▶ shows the carbon 1*s* X-ray Raman spectrum from an amorphous polycarbonate sample recorded using a 40-crystal X-ray Raman Johann spectrometer (Sokaras *et al.*, 2012 ▶) and a CSPAD-140k detector. This fixed-energy point-to-point focusing spectrometer was set at 6462.2 eV and the XPP’s diamond (111) monochromator was scanned to produce the energy-loss spectrum. A photodiode was used for normalization. This demonstrates the feasibility of XRS-based measurements at FELs.

Despite the successful performance of numerous XES experiments, even state-of-the-art multi-analyzer spectrometers can detect only up to a few percent of the total 4π solid angle emitted by an isotropic source. Moreover, imperfections of the curved X-ray Bragg optics result in further suppression of their efficiency/reflectivity. A promising approach, only possible with the high fluence achieved at FELs, is to stimulate the hard X-ray emission process, instead of using purely the spontaneous emission. In stimulated hard X-ray emission processes an incoming X-ray pulse ionizes a large number of transition metal ions, leaving a 1*s* core-hole behind and inverting the population to an excited state along its path. The subsequent decay results in stimulated 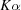 and 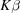 emission processes in the forward X-ray beam direction, and can lead to strong amplification gains along this well defined radiation direction. Both the amplification and the strong directionality of the amplified signal into a narrow beam in the forward direction could lead to dramatic gains in sensitivity and data collection time. Moreover, since the stimulated emission signal has a strong directionality, a single flat crystal could be positioned to diffract the signal onto a two-dimensional detector with the high-energy resolution characteristic of flat crystals and without the need of curved optics for high efficiency. The stimulated X-ray emission has already been demonstrated with soft X-rays at the LCLS (Rohringer *et al.*, 2012 ▶) and an experimental proof for hard X-rays is under way.

## 4. Conclusions   

Photon-in photon-out hard X-ray spectroscopy techniques are well established tools for studying the atomic and electronic structure of many materials. So far they have been largely performed at SR facilities for static studies. When performed at a FEL, the information content provided by these techniques makes them also powerful tools for studies of ultra-fast dynamics or other processes that rely on ultra-short ultra-bright X-ray pulses.

As discussed here, wavelength-dispersive spectrometers are very well suited for FEL applications as they allow for the collection of an entire X-ray spectrum pulse-by-pulse. We also discussed cases where monochromatic analyzers in the Rowland geometry are superior in terms of performance and efficiency. Over the last few years, both types of instrumentation have been successfully deployed at the LCLS to study ultra-fast and slow dynamics of chemical processes. In the latter case, the ultra-short FEL pulses were required to outrun radiation damage. These studies show that photon-in photon-out X-ray spectroscopy is already playing a central role in FEL-based research. We also showed that, depending on the sample type, the fluence of the incident FEL pulses can induce changes to the shape of XES spectra due to non-linear solid-state effects or multi-electronic excitations.

Future advances in FEL beam seeding and beam diagnostics, as well as analyzer, detector and sample delivery methods, will further enhance the scientific potential of photon-in photon-out X-ray spectroscopy. Finally, the development of new spectroscopy schemes, for example stimulated X-ray emission and other non-linear processes, will take full advantage of the unique properties of FELs.
